# Age-Related Differences in Hamstring Flexibility in Prepubertal Soccer Players: An Exploratory Cross-Sectional Study

**DOI:** 10.3389/fpsyg.2021.741756

**Published:** 2021-11-02

**Authors:** Federico Abate Daga, Marco Panzolini, Ruben Allois, Luca Baseggio, Samuel Agostino

**Affiliations:** ^1^Department of Clinical and Biological Sciences, University of Turin, Turin, Italy; ^2^School of Exercise and Sport Sciences, University of Turin, Turin, Italy

**Keywords:** biological maturation, muscles flexibility, stretching, youth soccer, kids

## Abstract

This study aimed to investigate the hamstring flexibility rate among prepubertal soccer players from U8 to U12 and the role of age and soccer years of practice on the course of hamstring flexibility. Six hundred eleven young Italian soccer players from a local soccer school in Turin were recruited for this research and assigned to each group according to their chronological age (U8 = 124 players; U9 = 130 players; U10 = 151 players; U11 = 89 players; and U12 = 120 players). Hamstring flexibility was measured using the Sit and Reach Test (SAR), while data analysis was run using a one-way analysis of variance (one-way ANOVA). Furthermore, Tuckey’s *post hoc* was used to determine differences among the classes of age. Finally, a bivariate ordinal regression analysis was used to evaluate a potential association between age categories and hamstrings flexibility. In addition, multivariable ordinal regression was used to analyze this relationship adjusted for the Body Mass Index (BMI). The one-way ANOVA showed significant differences in flexibility among groups (*F* = 32.76, *P* < 0.0001). Tuckey’s *post hoc* identified significant differences between U8 and U10 (*p* < 0.01; −2,39 cm of hamstring stretching), U8 and U11 (*p* < 0.05; −2.19 cm), U8 and U12 (*p* < 0.0001; −5.90), U9 and U12 (*p* < 0.0001; −4.98 cm), U10 and U12 (*p* < 0.0001; −3.5 cm), U11 and U12 (*p* < 0,001; −3.70 cm). In the bivariate ordinal regression analysis, there was a negative association between the age categories and hamstrings flexibility (*R*^2^ = 0.137; *p* < 0.0001). The association persisted in multivariable ordinal regression analysis adjusted for BMI (*R*^2^ = 0.138; *p* < 0.0001). This study underlines changes in hamstring flexibility across different age groups of prepubertal soccer players. The older and more experienced in soccer are less flexible than the younger, considering the hamstring muscles. Thus, appropriate stretching protocols should be included in prepubertal soccer training to avoid the risk of lead players to excess hamstring tightness.

## Introduction

“Hamstrings” is a term commonly used to refer to the muscles biceps femoris, semitendinosus, and semimembranosus, which form prominent tendons medially and laterally at the back of the knee. The hamstrings have a prevalence of fast-twitch fibers, and their main action is the flexion of the knee. For this reason, these muscles play a crucial role in several team sports such as soccer, which requires a lot of running, sprinting, rapid changes of direction, and jumping (Turner et al., 2014). However, they are often subjected to injuries: (Cejudo et al., 2019) it is estimated that 28% of soccer players experience hamstrings injuries at some point in their career (Ekstrand et al., 2016), with a risk of subsequent injury risk of 12–31% (Pagare et al., 2014). Therefore, hamstrings’ efficiency should be considered as a critical factor in soccer performance and injuries prevention (Le Gall et al., 2007; García-Pinillos et al., 2015; Medeiros et al., 2016). One essential characteristic of hamstring efficiency is flexibility (Medeiros et al., 2016), as it is a crucial quality in soccer performance, related to a lower incidence of injuries and a higher attendance to season matches (Witvrouw et al., 2003). Therefore, young soccer players’ development plan should include strategies for optimal development of the hamstrings muscles (Mayorga-Vega et al., 2016). Hamstrings origin from the ischial tuberosity, and thus affect posture and spine alignment during growth; (Cini et al., 2017) for example, an excessive shortening of these muscles increases the tension on the pelvis and, consequently, may produce changes in the spine morphology (López-Miñarro et al., 2012). Therefore, hamstring flexibility training should be included in youth soccer training programs, as they tend to become tighter throughout the biological maturation, it is unclear whether this is due to physical growth or it is influenced by daily routine and practice (Zakas et al., 2002). Even if hamstring injuries occurred at a lower incidence in youth players than adults, a study conducted among 623 young players of Barcelona Football Club revealed an incidence of damage estimate at 0.041 for biceps femoris and 0.061 for semitendinosus and semimembranosus every 1,000 h of soccer practice (Valle et al., 2018). Furthermore, early specialization is considered a risk factor for hamstring injuries (Difiori et al., 2014; Read et al., 2016). Despite this, only a few articles investigated muscle flexibility in young soccer players (Nikolaïdis, 2012; Nikolaidis et al., 2014; Cejudo et al., 2019), and hamstrings flexibility training in youth football is a debated topic. Indeed, most of the studies are conducted in adults or post-pubertal players, and thus soccer school coaches do not have enough information to implement hamstring flexibility training in the youth. A general trend toward the flexibility reduction overages has been reported in non-athlete population (De Oliveira Medeiros et al., 2013) but, to date, this has not been explored in soccer. Therefore, the aim of this study is to investigate the age-related differences in hamstring flexibility in soccer school players.

## Materials and Methods

The study was conducted in September and October 2021, before the beginning of the regular season. Participants were recruited from a soccer academy in the city of Turin, Italy, from different categories: Under 8 (i.e., players from 6 to 7 years of age), Under 9 (i.e., players from 8 to 9 years of age), Under 10 (i.e., players from 9 to 10 years of age), Under 11 (i.e., players from 10 to 11 years of age), and Under 12 (i.e., players from 11 to 12 years of age). The inclusion criteria were: being outfield players regularly involved in soccer training (i.e., 2–3 training sessions and one match per week) and having at least 1 year of experience in soccer training. The exclusion criteria were: being involved in systematic and specific stretching training programs in the last 6 months, history of hamstring or low back injuries in the previous 3 months, playing as goalkeepers, and a BMI over the 95 percentiles of the referred age (i.e., U8:19.20 kg/m^2^, U9: 19.77 kg/m^2^, U10:20.88 kg/m^2^, U11:22.06 kg/m^2^, and U12:22.67 kg/m^2^) (Weir and Jan, 2019; World Health Organization (WHO), 2020). This study was conducted in accordance with the Declaration of Helsinki and approved by the bioethical committee of the University of Turin, Italy (protocol number: 470603).

### Testing Procedure

All subjects were evaluated at their training center. The players were evaluated on one of their training days immediately before starting the training session before warming up, as warm-up procedures might influence hamstring flexibility (O’Sullivan et al., 2009). Anthropometric measurements were performed at the beginning of the testing session. Body mass was measured to the nearest 0.1 kg (Innoliving, Ancona, Italy), with the participants wearing their football equipment except for shoes and shin guards, while standing height was measured to the nearest 0.01 meter with a wall stadiometer (Metrica 23119, San Donato Milanese, Italy). Body weight and standing height were used to calculate the body max index (BMI; BMI = kg/m^2^).

The Sit and Reach Test (SAR) was performed with the subject sitting on the floor with his head, back, and hips against a wall, knees straight, legs together, and soles of the feet positioned flat against an SAR box (height: 30 cm, wide: 50 cm, and deep: 51 cm). The 0 cm mark of the measuring scale represented the starting point of the bar. The bar with the measuring scale was 80 cm long, running along the upper face of the SAR box. The place at which the feet contacted the box was 30 cm far from the starting point of the bar. The subject was required to extend the arms with palms down with index fingers in contact. Then, the subjects were asked to slowly bend forward to reach as far as possible while maintaining the knees extended and having their hands slide on the measuring scale placed on the box. The researcher should record the score and control that heels remained at the box and knees fully extended during the trial. Each player should perform only one attempt at their best. Allowing more than one attempt could convert a testing session into a flexibility training one. Therefore, only one try each player has admitted. The player was discharged and recalled to a new testing session on another training day in case of test failure. The SAR test is a straightforward way to investigate hamstring flexibility on large samples (Mayorga-Vega et al., 2014). Therefore, it was a functional test for this study.

Finally, to avoid the learning effect, all the soccer players completed a familiarization session to know the correct technical execution of the SAR test. Then, 1 week before the start of the study, each player tried to execute at least one time the SAR test.

### Statistical Analysis

Descriptive statistics [mean and standard deviation (SD), median and interquartile range (IQR)] was used to present participants characteristics and study outcomes. Age-related differences in hamstring flexibility were investigated using a univariate analysis of variance (ANOVA) with Tuckey’s *post hoc*. The significance level was set at α = 0.05. Bivariate ordinal regression analysis was used to evaluate a potential association between age and hamstrings flexibility. Multivariable ordinal regression was used to analyze the relationship between age and hamstrings flexibility adjusted for the Body Mass Index (BMI). R-Squared and associated *p*-values were reported for bivariate and multivariable analyses. All statistical analyses were conducted using SPSS Statistics (version 19.0; IBM Corp., Armonk, NY, United States).

## Results

**FIGURE 1:**
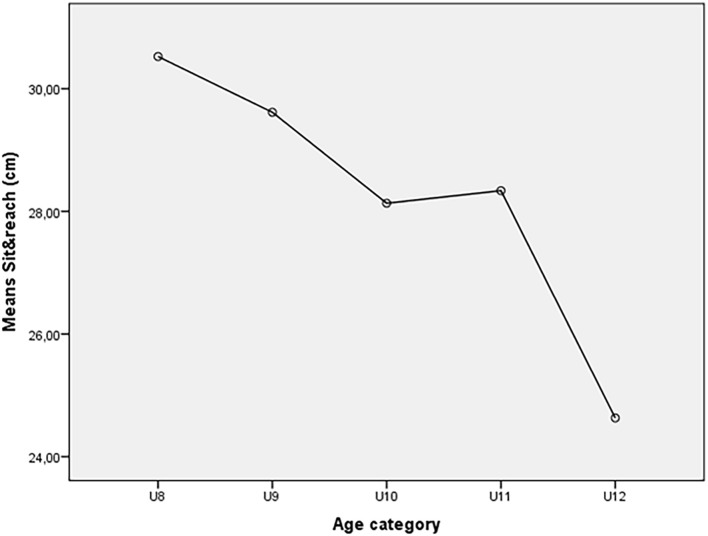
Representation of hamstring flexibility decrease across age. The most significant hamstring flexibility decline is between U11 and U12.

Six hundred eleven from five age categories (U8 = 124 players, height: 125.92 ± 5.52 cm, weight: 26.27 ± 3.90 kg, BMI: 16.53 ± 1.70 kg/m^2^, years of soccer school: 2 ± 1; U9 = 130 players, height 132.87 ± 8.84 cm, weight 29.49 ± 5.88 kg, BMI 16.89 ± 2.22 kg/m^2^, years of soccer school: 3 ± 1; U10 = 151 players, height 137.51 ± 5.88 cm, weight 34.54 ± 6.32 kg, BMI 18.16 ± 2.50 kg/m^2^, years of soccer school 4 ± 1; U11 = 89 players, height 140.90 ± 6.09 cm, weight 36.27 ± 5.78 kg, BMI 18.17 ± 2.22 kg/m^2^, years of soccer school: 5 ± 1; and U12 = 120 players, height 147.34 ± 6.09 cm, weight 39.12 ± 7.54 kg, BMI 17.93 ± 2.55 kg/m^2^, years of soccer school 6 ± 1) were enrolled in this study. The characteristics of the participants are presented in the Table 1. Sit and reach scores were: U8: 30.0 ± 5.4 cm, U9: 29.3 ± 5.5 cm, U10: 28.1 ± 5.6, U11: 28.3 ± 6.6, and U12: 24.4 ± 5.5 (Table 1). We observed statistically-significant differences in the SAR across the age groups: U8–U10 (*p* < 0.01, −2.4 cm), U8–U11 (*p* < 0.05, −2.2 cm), U8–U12 (*p* < 0.0001, −5.9 cm), U9–U12 (*p* < 0.0001, −5.0), U10–U12 (*p* < 0.0001, −3.5 cm), and U11–U12 (*p* < 0.001, −3.7 cm) (Table 2 and Figure 1). The bivariate regression model revealed a negative association between the age categories and hamstrings flexibility (*b* = −1.405, *R*^2^ = 0.137, *p* < 0.0001), and the association persisted if the model is adjusted for BMI (*R*^2^ = 0.138; *p* < 0.0001) (Table 3).

**TABLE 1:** Participants characteristics.

	Total (=611)	U8 (*n* = 124)	U9 (*n* = 130)	U10 (*n* = 151)	U11 (=86)	U12 (=120)
Age (years)	9 ± 1.59	7 ± 1	8 ± 1	9 ± 1	10 ± 1	11 ± 1
(mean ± SD)						
Weight (kg)	32.32 ± 7.29	26.27 ± 3.90	29.49 ± 5.88	34.54 ± 6.32	36.27 ± 5.78	39.12 ± 7.54
(mean ± SD)						
Height (cm)	135.51 ± 9.4	125.92 ± 5.52	132.87 ± 8.84	137.51 ± 5.88	140.9 ± 6.09	147.34 ± 6.03
(mean ± SD)						
BMI* (kg/m^2^)	17.47 ± 2.34	16.53 ± 1.70	16.89 ± 2.22	18.16 ± 2.5	18.17 ± 2.22	17.93 ± 2.55
(mean ± SD)						
Sit&Reach	28.63 ± 5.79	30.52 ± 5.30	30.00 ± 5.17	28.13 ± 5.56	28.34 ± 6.58	24.63 ± 5.15
(mean ± SD)						

	**U8 (*n* = 124)**	**U9 (*n* = 130)**	**U10 (*n* = 151)**	**U11 (=86)**	**U12 (=120)**	**Total**

Age (years)	7.00	8.00	9.00	10.00	11.00	9.00
(median)						
Weight (kg)	26.0 (4)	28.0 (6)	34.0 (9)	35.50 (8.25)	39.0 (8)	31.0 (10)
Median (IQR)						
Height (cm)	126.0 (7)	132.0 (8)	138.0 (7)	141.0 (9)	148.0 (8.5)	135.0 (12)
Median (IQR)						
BMI* (kg/m2)	16.08 (1.82)	16.28 (2.52)	17.82 (3.40)	17.74 (3.33)	17.6 (3.06)	16.89 (2.87)
Median (IQR)						

*Data are represented as mean ± standard deviation (SD) in the first part. On the opposite, in the second part, data are described as Median and Interquartile Range (IQR).*

*BMI, Body Mass Index.

**TABLE 2:** Description of the Tuckey’s *post hoc* performed among classes of age.

Tuckey’s *post hoc* among age categories			
		Mean difference (cm)	95% CI
U8	U9	0.91	(−0.99 to 2.81)
	U10	2.39**	(0.55 to 4.23)
	U11	2.19*	(0.06 to 4.31)
	U12	5.89*	(3.57 to 8.22)
U9	U10	148	(−0.33 to 3.30)
	U11	128	(−0.83 to 3.39)
	U12	4.99**	(2.68 to 7.29)
U10	U11	–0.21	(−2.25 to 1.84)
	U12	3.50**	(1.25 to 5.75)
U11	U12	3.71**	(1.21 to 6.19)

*p < 0.05; **p < 0.001.

**TABLE 3:** Representation of the bivariate (MODEL 1) and multivariable (MODEL 2) ordinal regression.

MODEL 1							MODEL 2						
			95% CI							95% CI			
	b	Beta coefficient	Lower	Upper	p	t		b	Beta coefficient	Lower	Upper	p	t
	40.786		38.22	43.35	< 0.001	31.232		41.80		38.19	45.40	< 0.001	22.765
AGE	–1.405	–0.370	–1.686	–1.125	< 0.001	–9.832	AGE	–1.37	−0.361	–1.664	–1.075	< 0.001	–9.133
BMI	−	−	−	−	−	−	BMI	–0.076	−0.031	–0.265	0.114	0.432	–0.786
R squared	0.137						R squared	0.138					

*In the multivariable ordinal regression. BMI did not affect the association between age categories and hamstrings flexibility.*

## Discussion

The present study shows that players of the U8 had the best performance in the SAR, while the U12 had the lowest performance. In addition, a reduction in hamstring flexibility of approximately 4 cm was detected between U11 and U12 players. Conversely, only 2 cm of hamstring flexibility reduction were identified between U8 (the youngest) and U11. The changes in hamstring flexibility seem to confirm that hamstring flexibility decreases with age (De Oliveira Medeiros et al., 2013; McKay et al., 2017).

Previous studies suggested that the decrease in hamstring flexibility with age can be due to biological changes, such as tendon stiffening, joint capsule changes, or muscle changes (Adams et al., 1999). Furthermore, it was demonstrated as tendon stiffening increases after resistance or power training (Seynnes et al., 2009). Thus, considering that repeated sprints, changes of directions, jumps, landing after jumping, or sudden deceleration from sprints characterizes soccer, it might be possible that these activities generate an early decrease of hamstring flexibility. Our outcomes seem to confirm this hypothesis underlining as hamstring flexibility is reduced across ages still from pre-puberty. On the opposite, hamstring flexibility was established as a critical factor for soccer performance in adults (Witvrouw et al., 2003) and youth (García-Pinillos et al., 2015). In fact, specific drills such as sprinting, jumping, agility, and kicking require good levels of hamstring flexibility to be efficient (García-Pinillos et al., 2015). Thus, considering its implication in soccer performance, flexibility training should not be underestimated from soccer school.

Moreover, the consistent significant difference in flexibility reduction detected from U11 to U12 is essential for this paper and should be evidenced. This stage is a sort of transition from childhood to puberty, where structural and physiological changes appear. The period between 12/13 and 15 years old corresponds to the Peak of Heigh Velocity (PHV). In this stage, the skeleton overgrows compared to muscles and tendons (Rumpf and Cronin, 2012). Therefore, muscle tightness rapidly increases, particularly around joints (Le Gall et al., 2007). This research evidenced a reduction of 4 cm in hamstrings flexibility from U11 to U12, certifying a double increase of hamstrings tightness compared with the one detected from U8 to U11, where all the players were still in the childhood stage. Thus, puberty might create an excess of hamstring tightness in soccer players that could be dangerous for muscle health, skill acquisition, and soccer performance.

This research identified an association between hamstring flexibility decrease and age categories, and this model explains the 14% variance. The remaining 86% could not be presented with the association between age categories and hamstrings flexibility reduction and might be related to other factors. For example, decreased hamstring flexibility could be due to altered neuro-dynamics (Kornberg and Lew, 1989), joint structure, viscoelastic properties, cross-sectional area, stretch tolerance (Magnusson et al., 1997), and type of training (Klinge et al., 1997). However, even if the effect is small, coaches, and team staff should carefully consider this association between age categories and hamstrings flexibility when setting soccer school training.

Last but not least, this study reveals some strengths and limitations. First of all, this study involved a large number of participants. A large sample usually guarantees more powerful findings. Therefore, the final message is commonly more robust. Furthermore, a quick and straightforward test like Sit and Reach is an efficient solution to screen a significant number of participants efficiently. However, on the other hand, such a considerable number of participants may lead to some limits. One of them may be identified in the impossibility of thoroughly assessing the lower back health before the evaluation. However, the hamstring flexibility assessment was performed using the SAR to repair this condition partially.

In addition, pelvis and spine mobility plays a crucial role in posterior chain flexibility assessment. High values of the pelvis and spine mobility can influence the hamstrings flexibility scores. However, this situation can be mechanically excluded from bending the upper body while seated. Thus, hamstring flexibility scores using SAR are not influenced by spine and pelvis mobility, and findings are more connected to muscle stretching potential. Secondly, physical growth may be another limitation of this research. Early or late maturation may affect muscles flexibility scores. In this research, no thorough assessment of physical growth was performed. As previously declared, large sample size is difficult to assess entirely due to logistics and practical organization. However, the inclusion criteria of this study indicated that only participants whose BMI did not exceed the 95th percentile of the referred age were eligible for this study. Therefore, findings were not affected by very early physical growth or obesity. This fact partially mitigates the limit of no physical growth assessment. Finally, future researches should investigate hamstring flexibility throughout an entire soccer season to analyze the impact of continuative soccer training on hamstring flexibility changes.

## Conclusion

In conclusion, this study highlighted the crucial difference in the hamstring flexibility measurement among prepubertal male soccer players of different ages. In addition, players of the older age category have lesser hamstring flexibility measurement compared to younger ones. Thus, hamstring tightness is highly enhanced in players belonging to the U12. Therefore, hamstring flexibility schedules must be empowered in the so-called “pubertal stage” to avoid the risk of hamstring injuries exposure during biological maturation.

## Data Availability Statement

The original contributions presented in the study are included in the article/supplementary material, further inquiries can be directed to the corresponding author/s.

## Ethics Statement

The studies involving human participants were reviewed and approved by the University of Turin Bioethical Committee (Comiato bioetico di Ateneo), University of Turin, Turin, Italy. Written informed consent to participate in this study was provided by the participants’ legal guardian/next of kin.

## Author Contributions

FA contributed to the manuscript’s conception and design and supervised the experimental phase. SA, LB, and MP conducted the experiment, performed the calculations, and organized the tables and graphs. FA wrote the manuscript with input from all authors. All authors read and approved the final version of the manuscript. Finally, RA assisted all authors during the manuscript writing and observed reviewer’s recommendations and manuscript modification.

## Conflict of Interest

The authors declare that the research was conducted in the absence of any commercial or financial relationships that could be construed as a potential conflict of interest.

## Publisher’s Note

All claims expressed in this article are solely those of the authors and do not necessarily represent those of their affiliated organizations, or those of the publisher, the editors and the reviewers. Any product that may be evaluated in this article, or claim that may be made by its manufacturer, is not guaranteed or endorsed by the publisher.
